# Wearable devices in the management of hypertension: Navigating the intersection between digital advancement and evidence-based practice

**DOI:** 10.1016/j.clinme.2026.100595

**Published:** 2026-05-14

**Authors:** Abubaker Eltayeb, Ayesha Ahmed, Ajay Gupta

**Affiliations:** William Harvey Heart Centre, Queen Mary University of London, EC14JN London, UK

**Keywords:** Hypertension, Blood pressure variability, Cuffless devices, Validation, Cardiovascular mortality

## Abstract

Hypertension remains the leading cause of premature mortality. Its effective management is hindered by sub-optimal monitoring from intermittent measurement of blood pressure, which often lacks important prognostic data such as patterns of blood pressure variability. This article provides a summary of the technologies used in wearable devices, an evaluation of their emerging role in the management of hypertension, and current challenges to their utility. Unlike traditional cuff-based devices, cuffless technology utilises range of sensors and physiological principles, including peripheral pulse wave characteristics and photoplethysmography, together with algorithms to estimate blood pressure. Cuffless devices are unobtrusive and can be worn continuously, providing longer-term data. As such, they can bridge gaps in identifying and monitoring high-risk hypertension profiles, sleep periods data, dipping status and blood pressure variability, which can guide individualised clinical decision making. However, due to rapid innovation and technological heterogeneity and associated lack of appropriate validation frameworks, there remain significant concerns on the accuracy and reliability of cuffless wearables for clinical use. Despite their potential and market availability, professional society guidelines do not recommend the use of cuffless devices for clinical decision making until more robust and complete real-world evidence is available.

## Highlights

• Out-of-office blood pressure measurements are essential for accurate diagnosis and effective management of hypertension.
• Cuffless wearable blood pressure devices do not directly measure blood pressure, but they estimate it from surrogate signals.
• Current evidence suggests shortfalls in the validation of cuffless devices, including inaccuracies in estimates of night-time blood pressure.
• Clinical guidelines recommend against using cuffless devices in practice while validation efforts continue.

## Graphical Abstract

**Figure:**
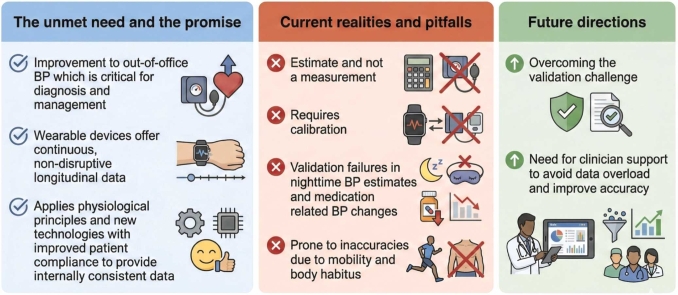
The role of wearable devices in the management of hypertension.

## Introduction

High blood pressure (BP) remains a leading preventable risk factor for cardiovascular disease (CVD) morbidity and mortality globally, with an estimated 10.8 million cardiovascular deaths and 11.3 million deaths overall in 2021.^1^ For early diagnosis and effective management of hypertension, accurate measurement and monitoring of BP are essential. Guidelines provide standards for accurate cuff-based office and home BP measurement; however, these only provide snapshot measures of BP, with a number of limitations such as the inability to independently differentiate between white-coat hypertension and masked hypertension, and are reliant on proper technique.^2^, ^3^, ^4^, ^5^, ^6^

Ambulatory BP monitoring (ABPM) over 24 h offsets some of these limitations, identifying high-risk phenotypes and nocturnal dipping profiles, and is strongly predictive of clinical outcomes. However, ABPM is expensive, resource intensive, often poorly tolerated and unable to provide longer-term BP trends and variability patterns.^4^, ^7^ The fundamental challenge in blood pressure monitoring today is the shift from episodic to continuous measurements providing longitudinal data, and wearable devices, particularly those employing cuffless technologies, such as smart watches, bracelets and rings, have appeared as an increasingly available user-friendly alternative for near continuous BP measurement.^8^

See Table 1 for a comparison between currently available BP-measuring modalities.

**Table 1:** Comparison of blood pressure measurement modalities.

Feature	Office BP	Home BP	Ambulatory BP	Cuffless devices
Principle	Auscultatory/oscillometric	Oscillometric	Oscillometric	Sensor-based algorithm estimation
Measurement	Static in clinic	Static at home	24 h, automated, in intervals	Continuous, intermittent
Key data	Few clinic measurements	Mean home BP	Means of 24 h, wake periods, and sleep periods, dipping, and SD^a^	Long-term mean BP, TTR^b^, BPV^c^, position, sleep periods BP
Advantages	Clinician supervised	Inexpensive, removes white-coat and masked hypertension effects, longitudinal data	Removes white-coat and masked hypertension effects, nocturnal BP, dipping profile	Convenience to patients, high frequency of data, measures TTR^b^
Limitations	No nocturnal data, white-coat and masked hypertension	Relies on patient’s technique and adherence, no sleep period data, can induce anxiety	Limited to 24 h, resource intensive, may be poorly tolerated, sleep disruption	Requires calibration, need for validation, failure to track medication changes, data overload
Guideline status	Recommended for screening	Recommended for diagnosis and management	Gold standard for diagnosis and prognosis	Not yet recommended for clinical use

a SD, standard deviation;

b TTR, time in target range;

c BPV, blood pressure variability.

## New wearable technologies and their mechanisms

Wearable BP-measuring devices can broadly be classified into either cuff-based or cuffless. Miniaturised oscillometric devices, applied to the wrist or finger, retain the traditional cuff oscillometric principle but in a compact form. Commercial examples, such as HeartGuide, marketed primarily in Asian healthcare systems, improve portability and offer performance close to that of validated upper-arm measurements, which still require cuff inflation, and they provide intermittent rather than continuous BP data.^9^

Cuffless BP monitoring leverages non-invasive sensor technologies and physiological modelling to provide near-continuous data. The most established approach utilises photoplethysmography (PPG), often synchronised with electrocardiography (ECG) to capture volumetric and electrical changes in the cardiac cycle. Other emerging modalities include tonometry, bioimpedance and multimodal sensor fusion, which analyse changes in thoracic or limb electrical properties alongside mechanical signals. BP is estimated by determining physiological parameters such as pulse wave velocity, pulse arrival time, pulse transit time, and/or by examining wave morphology in pulse wave analysis. through algorithms, employing machine learning and deep learning, which can mitigate issues like motion artefact.^8^, ^10^, ^11^, ^12^, ^13^, ^14^ In most cases, cuffless devices require calibration, initially and then periodically; where the user enters a BP value self-measured using a validated oscillometric cuff-based device into the wearable to allow it to personalise its model and essentially track BP changes relative to the latest calibration. It is important to emphasise that these devices provide estimates of BP, and their accuracy is dependent on a stable relationship between the used technology, cardiac and vascular parameters, and the BP.^12^, ^13^, ^15^

See Table 2 for a comparison between those technologies.

**Table 2:** Comparison of various wearable technologies for blood pressure measurement, and the current stage of clinical validation stage.

Technology category	Measurement principle	Cuff required	Measurement continuity	Calibration required	Accuracy vs reference cuff	Key strengths	Key limitations	Clinical validation status
Miniaturised cuff- based oscillometric	Oscillometric BP measurement using an inflatable cuff	Yes	No	No (standard cuff validation)	High (closest to upper-arm cuff)	Clinically validated principle; good accuracy	Bulky; cuff inflation; not continuous	Validated against international standards (AAMI/ESH/ISO); limited wearable-specific validation
Pulse transit time (PTT) / pulse arrival time (PAT)	Time delay between cardiac event and peripheral pulse	No	Yes	Yes (individual, repeated)	Variable	Cuffless; continuous monitoring potential	Calibration drift; sensitive to vascular tone	Partial validation in controlled studies; insufficient evidence for routine clinical use
PPG waveform analysis with machine learning	Pulse waveform feature extraction with algorithms	No	Limited	Yes (periodic cuff calibration)	Moderate (trend-level)	High user acceptability; scalable consumer use	Calibration dependence; limited diagnostic accuracy	Limited validation; acceptable for trend monitoring only, not diagnostic use
Bioimpedance and multimodal sensor fusion	Electrical impedance changes with multimodal signals	No	Yes	Likely (method dependent)	Promising but unproven	Fully cuffless; continuous monitoring potential	Limited validation; prototype stage	Early feasibility and pilot studies only; no large- scale clinical validation

## Clinical utility and validation challenges

New wearable technologies offer promising roles in the management of hypertension. A minimal-burden alternative to cuff-based ambulatory BP monitoring, wearable devices have the potential to enable screening diagnoses and management of hypertension at scale. The ability to alert a user to changing BP patterns fosters engagement, which can motivate/sustain medication adherence and healthy behaviours. Medication non-adherence remains a major driver of treatment failure. Some studies suggest improved active patient engagement and medication adherence with interventions using digital health technologies.

Wearable devices are uniquely positioned to identify phenotypes such as masked hypertension, with characteristically normal office BP readings, and to provide continuous data on nocturnal BP as opposed to the single 24-h ABPM which is limited by a single-night set of readings, which can improve detection of high-risk nocturnal hypertension and morning BP surges. In addition, continuous data on BP supplemented with data changes on patient behaviours, lifestyle and activity can enhance and guide timely management of medications in cases of blood pressure variability and change to BP control. Moreover, new wearable technologies offer an opportunity for the integration of the large volume of data they provide with the healthcare system utilising new advances in generative artificial intelligence (GenAI). This can potentially provide clinicians with contextualised summaries, streamline clinical workflow, and reduce clinician inertia.^8^, ^10^, ^12^, ^16^, ^17^ The ability for data sharing with healthcare providers systems can enable prompt and personalised clinical decision making. However, there are key challenges to address before the clinical utility of wearable devices can be fully realised, including:

1. Accuracy and validation – across population groups, national regulatory standards, for dynamic states of comparison and especially post-treatment titration
2. Patient access and literacy
3. Data communication and integration into electronic health records or care pathways – standardising BP data summaries with secure transferring
4. Association with clinical outcomes to allow adoption in existing/modified hypertension guidelines.^18^

With rapid innovation in cuffless technology outpacing the development of validation and regulatory standards, the translation of their promise of accurate near-continuous BP monitoring into a clinical reality remains a difficult challenge. Validation of cuffless wearable devices is expected to demonstrate the devices’ ability to track physiological changes and provide accurate estimates of BP in dynamic states, such as during sleep and physical activity, which make existing cuff devices’ validation protocols inappropriate as they provide a static-state comparison against a reference cuff device.^11^, ^19^

Some validation studies suggest that mean BP estimates by wearable devices fall within a narrow range of difference to a cuffed reference device measurement. A meta-analysis aggregated data from 16 studies and reported a pooled mean BP bias of 3.42 mmHg (95% CI −2.71–9.01) for systolic BP and 1.16 mmHg (95% CI: −1.26–3.58) for diastolic BP. However, studies reported on cuffless devices employing a variety of sensing methods and used a range of reference devices and validation techniques, resulting in a large degree of heterogeneity. Authors did not report on device measurement precision for instance with standard deviation^8^ which forms an additional criterion in conventional validation protocols.

One study using a static validation protocol suggested that a commonly available PPG-based wrist device could accurately estimate BP in various positions with a mean difference of 0.46 ± 7.75 mmHg for systolic BP,^20^ while a later study, validating the same device against ABPM and home BP, highlighted systematic failures in night-time estimates as well as in tracking medication-related BP changes.^21^

Such validation challenges, including night-time BP and medication response tracking, and establishing reliable performance during activities of daily life, coupled with the growing availability and use of cuffless wearable devices by patients, necessitate the importance of understanding and bridging gaps in accuracy and efficacy before wearable devices can be safely used in clinical practice.^19^, ^22^, ^23^, ^24^

## Conclusions and future horizons

Existing hypertension diagnosis and management guidelines are driven by evidence that has examined clinical outcomes as they relate to cuffed blood pressure. While there is a clear need for improved out-of-office BP monitoring and identification of high-risk phenotypes, and the theoretical solution presented by wearable devices with continuous longitudinal data is compelling, current evidence fails to demonstrate their accuracy and utility for clinically meaningful monitoring and guiding antihypertensive therapy.

The future role of wearable devices is promising but requires robust validation using appropriate updated frameworks, across the spectrum of age, sex, ethnicity and health states such as pregnancy and frailty.^22^ Coupled with artificial intelligence and machine learning for interpretation of real-time BP data, wearables may be integrated in clinical decision-support systems that target treatment inertia and medication adherence and facilitate self-monitoring of control indices such as time in target range. Until evidence for such clinical translation is generated, the diagnosis and management of hypertension rely exclusively on validated cuff-based devices.

## CRediT authorship contribution statement

**Ajay Gupta:** Writing – review & editing, Supervision, Project administration, Methodology, Conceptualization. **Abubaker Eltayeb:** Writing – review & editing, Writing – original draft, Methodology, Data curation, Conceptualization. **Ayesha Ahmed:** Writing – review & editing, Writing – original draft, Methodology, Data curation.

## Funding

This research did not receive any specific grant from funding agencies in the public, commercial or not-for-profit sectors.

AG’s time is part-funded by a National Institute for Health and Care Research (NIHR) grant (NIHR202116).

## Declaration of generative AI and AI-assisted technologies in the writing process

During the preparation of this work, the authors used EndNote 2025.1 for referencing and citations. After using this tool, the authors reviewed and edited the content as needed and take full responsibility for the content of the publication.

## Declaration of competing interest

The authors declare that they have no known competing financial interests or personal relationships that could have appeared to influence the work reported in this paper.
